# Rescue of myocytes and locomotion through *AAV2/9-2YF* intracisternal gene therapy in a rat model of creatine transporter deficiency

**DOI:** 10.1016/j.omtm.2024.101251

**Published:** 2024-04-23

**Authors:** Gabriella Fernandes-Pires, Marcelo Duarte Azevedo, Marc Lanzillo, Clothilde Roux-Petronelli, Pierre-Alain Binz, Cristina Cudalbu, Carmen Sandi, Liliane Tenenbaum, Olivier Braissant

**Affiliations:** 1 Service of Clinical Chemistry, University of Lausanne and Lausanne University Hospital, Lausanne, Switzerland; 2 Laboratory of Cellular and Molecular Neurotherapies, Clinical Neurosciences Department, University of Lausanne and Lausanne University Hospital, Lausanne, Switzerland; 3 Centre d'Imagerie Biomedicale (CIBM), Ecole Polytechnique Fédérale de Lausanne (EPFL), Lausanne, Switzerland; 4 Animal Imaging and Technology, Ecole Polytechnique Fédérale de Lausanne (EPFL), Lausanne, Switzerland; 5 Brain Mind Institute, Ecole Polytechnique Fédérale de Lausanne (EPFL), Lausanne, Switzerland

**Keywords:** creatine transporter deficiency, SLC6A8, gene therapy, adeno-associated virus, brain, muscle, locomotion

## Abstract

Creatine deficiency syndromes (CDS), caused by mutations in *GATM* (AGAT), *GAMT*, and *SLC6A8*, mainly affect the central nervous system (CNS). CDS show brain creatine (Cr) deficiency, intellectual disability with severe speech delay, behavioral troubles, epilepsy, and motor dysfunction. AGAT/GAMT-deficient patients lack brain Cr synthesis but express the Cr transporter SLC6A8 at the blood-brain barrier and are thus treatable by oral supplementation of Cr. In contrast, no satisfactory treatment has been identified for Cr transporter deficiency (CTD), the most frequent of CDS. We used our *Slc6a8*^*Y389C*^ CTD rat model to develop a new *AAV2/9-2YF*-driven gene therapy re-establishing the functional Slc6a8 transporter in rat CNS. We show, after intra-cisterna magna *AAV2/9-2YF-Slc6a8-FLAG* vector injection of postnatal day 11 pups, the transduction of Slc6a8-FLAG in cerebellum, medulla oblongata, and spinal cord as well as a partial recovery of Cr in these brain regions, together with full prevention of locomotion defaults and impairment of myocyte development observed in *Slc6a8*^*Y389 C/y*^ male rats. While more work is needed to correct those CTD phenotypes more associated with forebrain structures, this study is the first demonstrating positive effects of an *AAV*-driven gene therapy on CTD and thus represents a very encouraging approach to treat the so-far untreatable CTD.

## Graphical abstract

**Figure:**
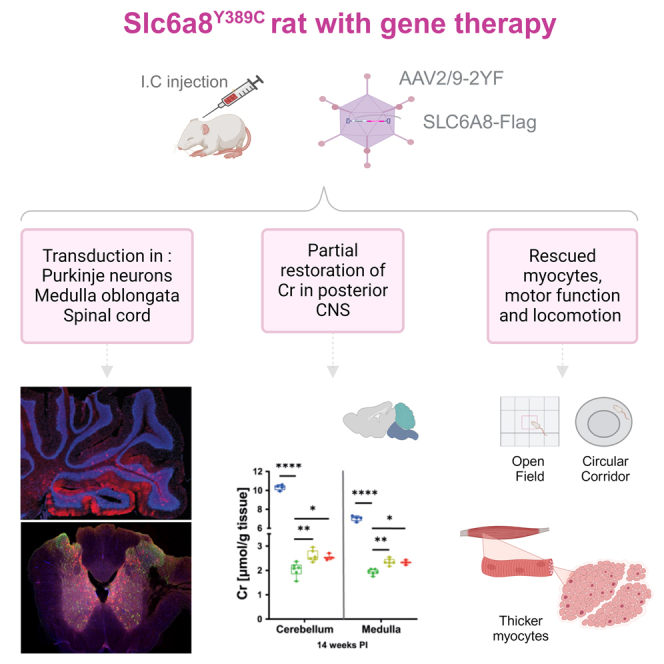

## Teaser

Braissant and colleagues show the AAV9-driven rescue of locomotion and myocytes in a rat model of creatine transporter deficiency through partial restoration of creatine in hindbrain and spinal cord.

## Introduction

Creatine (Cr, or α-N-methyl-guanidino-acetic acid) plays essential roles in ATP regeneration and transport of high-energy phosphates within cells.^1^^,^^2^^,^^3^ In humans, half of the daily Cr needs are obtained through diet, the other half being synthesized by a two-step pathway involving arginine:glycine amidinotransferase (AGAT/EC 2.1.4.1) and guanidinoacetate methyltransferase (GAMT/EC 2.1.1.2). Cells take up Cr by a specific transporter, SLC6A8 (also known as CT1, CRT, CRTR, or CreaT) belonging to the solute carrier family 6, with the co-transport of two Na^+^ and one Cl^−^.^4^^,^^5^^,^^6^

Deficit in Cr synthesis or transport leads to creatine deficiency syndromes (CDS). AGAT and GAMT deficiencies affect males and females comparably as they are autosomal recessive. Cr transporter deficiency (CTD) is X-linked, male SLC6A8-deficient patients being affected while female heterozygous SLC6A8-deficient patients can be completely normal (often not knowing that they carry a mutated *SLC6A8* gene) or present a wide phenotypic variability from mild to severe disease, due to the random inactivation of the X chromosomes.^7^^,^^8^^,^^9^^,^^10^ These three diseases are characterized by the absence, or very strong decrease, of Cr in the brain when measured by ^1^H-magnetic resonance spectroscopy (^1^H-MRS).^11^^,^^12^^,^^13^^,^^14^

CTD (OMIM: 300352) patients develop neurological symptoms such as intellectual and developmental delay (ID/DD) and severe problems of speech acquisition, seizures, as well as behavioral and movement disorders.^9^^,^^15^^,^^16^

While AGAT and GAMT deficiencies can be treated by Cr supplementation leading to neurological improvement, no satisfactory treatment has been found so far for CTD, its current treatment strategies being limited to managing seizures and behavioral problems.^17^^,^^18^^,^^19^^,^^20^^,^^21^^,^^22^ Some female patients, however, can benefit from Cr supplementation, as they probably keep some residual SLC6A8 activity at the blood-brain barrier (BBB) due to their SLC6A8 heterozygous status.^23^

To better understand CTD and develop new treatment strategies, different *in vivo* CTD models have been generated: (1) ubiquitous gene deletion knockout (KO) mice through *Slc6a8* exons removal,^24^^,^^25^^,^^26^ including (2) through tamoxifen induction,^27^ (3) brain-specific KO mice through CRE-induced *Slc6a8* exons removal,^28^^,^^29^^,^^30^^,^^31^^,^^32^ and (4) our recently described knockin (KI) *Slc6a8*^*Y389C*^ rat model bearing one of the single-nucleotide mutations described in CTD patients.^33^ All these CTD models present the CTD-characteristic brain Cr deficiency and decreased body weight, as well as cognitive deficits, stereotypical behavioral alterations, or impaired memory performance. However, only two of them show some of the motor dysfunction phenotypes of CTD.^26^^,^^34^ In particular, our *Slc6a8*^*Y389C*^ rat shows mild impaired locomotor function with reduction of muscular mass and thinner myocytes.^33^^,^^34^

We used our *SLC6A8*^*Y389C*^ KI rat to develop a new adeno-associated virus (AAV) gene-therapy strategy. We present the *AAV* central nervous system (CNS) transduction of *Slc6a8*^*Y389 C/y*^ KI males (mKI) with a functional Slc6a8-FLAG transporter. Intra-cisterna magna (IC)-injected mKI showed a significant Slc6a8-FLAG transduction in cerebellum, medulla oblongata, and spinal cord, together with the full prevention of the locomotor deficit and myocyte thinness observed in non-injected *Slc6a8*^*Y389C/y*^ mKI.

## Results

### Transduction of the Slc6a8-FLAG protein in cerebellum, medulla oblongata, and spinal cord of *AAV2/9-2YF-Slc6a8*-injected mKI

**Figure 1:**
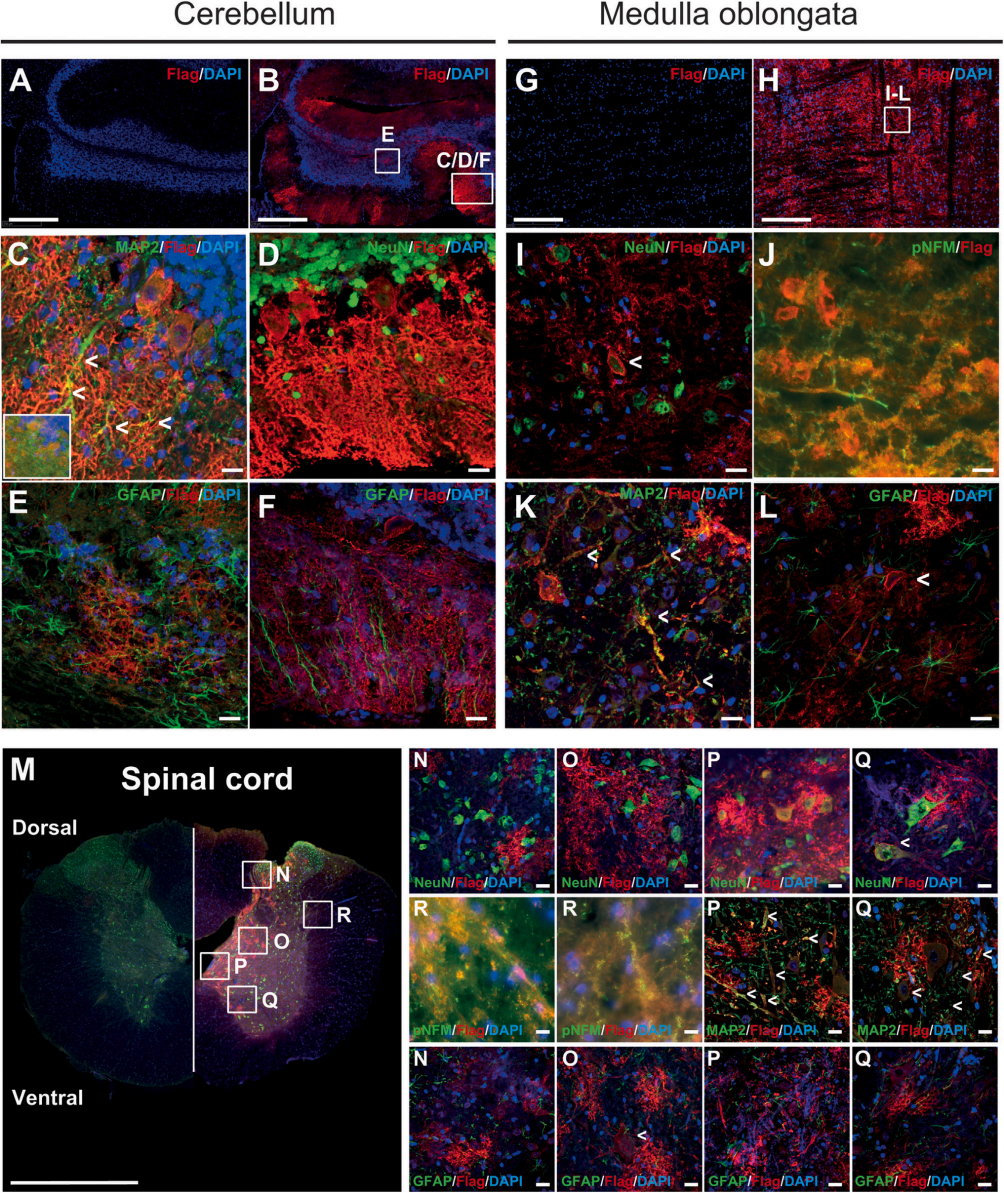
Transduction of the Slc6a8-FLAG protein in CNS of *AAV2/9-2YF-Slc6a8*-injected mKI rats at 14 weeks PI Representative images of immunostaining for Slc6a8-FLAG in different regions of the brain: cerebellum of (A) non-injected mKI or (B–F) *AAV2/9-2YF-Slc6a8*-injected mKI, medulla oblongata of (G) non-injected mKI or (H–L) *AAV2/9-2YF-Slc6a8*-injected mKI; (C and E) granular layer of cerebellum; (D and F) molecular layer of cerebellum; and (I–L) medulla oblongata. (M) Cross-section of the spinal cord; left, non-injected mKI; right, *AAV2/9-2YF-Slc6a8*-injected mKI. (N) lamina I/II, (O) lamina IV/V, (P) lamina X, and (Q) lamina VII of spinal cord, (R) axons of dorsal root of a spinal nerve. Slc6a8-FLAG in red; NeuN, GFAP, MAP2 or pNFM in green; DAPI in blue. Scale bar for overview of cerebellum, medulla, and spinal cord: 250 μm. Scale bar for details of cerebellum, medulla, and spinal cord: 20 μm.

While *AAV2/9-2YF-Slc6a8* vector IC injection did not lead to an efficient Slc6a8-FLAG protein transduction in more anterior parts of the brain (olfactory bulbs, cortex, hippocampus, diencephalon, midbrain; Figure S1; Table 1), a widespread transduction of Slc6a8-FLAG was observed in cerebellum, pons/medulla oblongata, and spinal cord of injected mKI at both 5 (Figure S2) and 14 weeks post injection (PI) (Figure 1; Table 1). In non-injected mKI controls, no Slc6a8-FLAG protein could be detected, as expected (Figures 1A, 1G, and 1M). As the transduced Slc6a8-FLAG protein is localized on the cell membrane (Figure S3) and often expressed very far from the cell body, preliminary experiments in male wild-type (mWT) rats with control *AAV2/9-2YF* vectors transducing the fluorescent reporter proteins enhanced green fluorescent protein (EGFP) and mCherry were also used under the same conditions as *AAV2/9-2YF-Slc6a8* to identify the transduced brain cells, and they showed the same widespread transduction of EGFP and mCherry in hindbrain and spinal cord as rats transduced with *AAV2/9-2YF-Slc6a8* at both 5 and 14 weeks PI (Figure S4).

**Table 1:** Proportion of *AAV2/9-2YF-Slc6a8-FLAG*-transduced neurons in the *Slc6a8*^*Y389C*^ rat CNS

Forebrain	olfactory bulbs, cortex, basal ganglia, hippocampi, thalamus, hypothalamus	<1%
Midbrain	tectum, tegmentum, cerebral peduncles	<1%
Hindbrain∗	Cerebellum∗	
	molecular layer	<1%
	Purkinje cells∗	
	lobules 1–9a	<1%
	lobule 9b**∗**	1%–10%∗
	lobule 10∗	56.6% ± 4.8% (counting: 4; 76–220 cells/counting)∗
	granular layer	<1%
	cerebellar central nuclei	<1%
	Pons/medulla oblongata∗	high density of transduced axons and dendrites∗
Spinal cord∗	lumbo-sacral spinal cord∗	56.9 ± 2.2% (neuronal soma)∗ (counting: 4; 110–140 cells/counting)∗ + high density of transduced dendrites and axons, including from sensory and motor neurons∗

Highly transduced regions indicated by asterisks.

In cerebellum, the most targeted cells were Purkinje neurons showing a strong expression of Slc6a8-FLAG on their dendritic tree (Figures 1B, 1C, 1D, and 1F; including co-stained dendrites with microtubule-associated protein 2/MAP2 Figures 1C, S2C, and S2D). This was particularly true in lobules 9 and 10 of cerebellum, with 1%–10% and 56.6% of transduced Purkinje neurons, respectively (Table 1). In the molecular layer, no co-staining was observed between Slc6a8-FLAG and neuronal nuclear protein (NeuN), phosphorylated medium-weight neurofilament (pNFM), or glial fibrillary acidic protein (GFAP), both at 5 and 14 weeks PI (Figures 1C, 1E, 1F, S2C, S2D), suggesting that no other neurons, or axons (including parallel fibers of granular neurons), or astrocytes (Bergman glia fibers) were transduced. In the granular layer, injected animals presented a few transduced granular neurons at 14 weeks PI, which were co-stained with NeuN, but no cell was observed co-stained with GFAP (Figures 1E, S2A, and S2B). Finally, some neurons were also transduced in the cerebellar nuclei (Slc6a8-FLAG co-localization with NeuN, but not with GFAP; Figures S2E and S2F). AAV-induced EGFP and mCherry transduction showed the same results (Figures S4A–S4D). Medulla oblongata also showed a strong neuronal transduction (co-staining with NeuN), including in axons and dendrites (co-staining with pNFM and MAP2 respectively) but not in astrocytes (co-staining with GFAP) (Figures 1H–1L, S2G, S2H, and S3B). AAV-induced EGFP and mCherry transduction also showed a strong axonal expression (Figures S4E–S4H).

As IC injection of *AAV9* vectors in *in vivo* animal models is known to transduce spinal cord,^35^^,^^36^ and, as SLC6A8 is highly expressed in motor neurons of somatic motor and visceromotor cranial nerve nuclei as well as in ventral horn of spinal cord,^37^ we analyzed the spinal cord for Slc6a8-FLAG transduction. Slc6a8-FLAG was expressed in the spinal cord of injected mKI at both 5 (Figures S2I–S2L) and 14 weeks PI (Figures 1M–1R). In laminas I, II, and X, Slc6a8-FLAG-positive cells were co-stained with MAP2, but not with NeuN nor GFAP, suggesting an expression of Slc6a8-FLAG in dendrites of neurons (Figures 1N and 1P, see white arrows; Figure S2J). In laminas IV, V, and VIII, Slc6a8-FLAG-positive cells were co-stained with NeuN and MAP2, but not with GFAP (Figures 1O and 1Q, see white arrows; Figure S2K), also suggesting a neuronal expression of Slc6a8-FLAG, with 56.9% of NeuN-positive neuronal soma shown transduced (Table 1). Axons in dorsal (Figure 1R) and ventral (data not shown) roots of the spinal cord also expressed the Slc6a8-FLAG transporter (co-stained with pNFM). AAV-induced EGFP and mCherry transduction showed the same results (Figures S4I–S4J).

**Figure 2:**
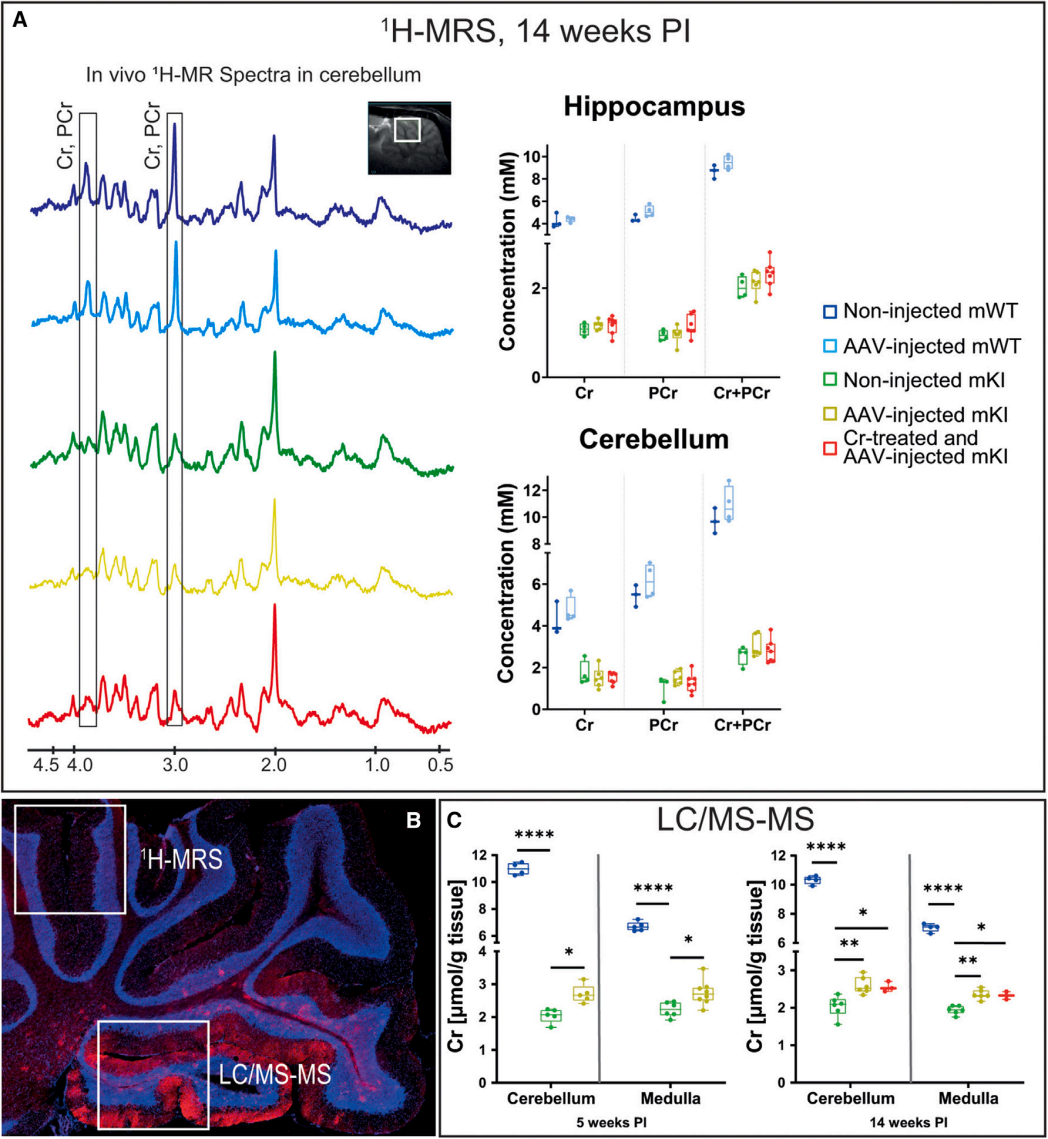
Partial restoration of brain Cr in cerebellum and medulla of *AAV2/9-2YF-Slc6a8*-injected mKI rats (A) Left: representative 9.4 T ^1^H-MRS in the dorsal lobules 6 and 7 of cerebellum of non-injected mWT and mKI, as well as *AAV2/9-2YF-Slc6a8*-injected mKI, plus or minus Cr supplementation, at 14 weeks PI, showing no recovery of Cr after *AAV2/9-2YF-Slc6a8-FLAG* injection. Right: measure of Cr and phosphocreatine (PCr) by ^1^H-MRS in hippocampus and dorsal lobules 6 and 7 of cerebellum, showing no significant recovery of Cr and PCr in *AAV2/9-2YF-Slc6a8*-injected mKI rats. Three non-injected mWT, four *AAV2/9-2YF-Slc6a8*-injected mWT, four non-injected mKI, six *AAV2/9-2YF-Slc6a8*-injected mKI, and seven Cr-supplemented and *AAV2/9-2YF-Slc6a8*-injected mKI. (B) Representative histological immunostaining of Slc6a8-FLAG in the cerebellum of *AAV2/9-2YF-Slc6a8*-injected mKI, showing a strong transduction of Slc6a8-FLAG in the posterior lobules 9 and 10 of cerebellum. White boxes represent where ^1^H-MRS or LC/MS-MS were performed. Red, Slc6a8-FLAG; blue, DAPI. (C) Significant increase of Cr levels in posterior lobules 9 and 10 of cerebellum and medulla oblongata in *AAV2/9-2YF-Slc6a8*-injected mKI, plus or minus Cr supplementation at 5 weeks PI (left graphs) and 14 weeks PI (right graphs) (μmol/g tissue). For 5 weeks PI, four non-injected mWT, five non-injected mKI, five *AAV2/9-2YF-Slc6a8*-injected mKI; for 14 weeks PI, four non-injected mWT, six non-injected mKI, six *AAV2/9-2YF-Slc6a8*-injected mKI, and three Cr-supplemented and *AAV2/9-2YF-Slc6a8*-injected mKI. Two-way ANOVA with *post hoc* Tukey test, ∗*p* < 0.05, ∗∗*p* < 0.01, ∗∗∗∗*p* < 0.0001.

Within the whole brain and spinal cord, no oligodendrocytes could be identified as expressing the transduced Slc6a8-FLAG transporter (co-staining with myelin basic protein [MBP]). No Slc6a8-FLAG-positive cell could be observed either with the characteristic morphology of microglia, whether dendritic or reactive. Finally, microcapillary endothelial cells (MCECs) at BBB did not appear transduced either, with the functional observation that Cr supplementation of *AAV2/9-2YF*-injected mKI did not lead to any further Cr replenishment (see below, Figure 2C). In conclusion, every brain cell or cellular process in which the *AAV2/9-2YF*-transduced Slc6a8-FLAG protein could be identified was of neuronal origin.

### mKI brain Cr under *AAV2/9-2YF-Slc6a8* injection: No replenishment of Cr in the forebrain but partial Cr correction in posterior cerebellum and medulla oblongata

CTD is characterized by a strong reduction of brain Cr, measured *in vivo* by ^1^H-MRS, which was also observed in *Slc6a8*^*Y389C/y*^ mKI.^33^ While this study confirmed the strong cerebral Cr decrease in non-injected mKI (9.4T ^1^H-MRS in hippocampus and superior lobes of cerebellum; Figures 2A and 2B), we did not observe any significant correction (i.e., increase) of brain Cr in these same regions of injected mKI at 5 (data not shown) and 14 (Figures 2A and 2B) weeks PI, whether supplemented or not with Cr.

However, the brain structures measured by ^1^H-MRS were regions where the Slc6a8-FLAG protein transduction could not be observed or only in very rare cells (hippocampus and dorsal lobules 6 and 7 of cerebellum; Figure 2B; Table 1), most probably explaining the non-recovery of Cr observed by ^1^H-MRS in these regions. We therefore decided to measure Cr by liquid chromatography coupled to tandem mass spectrometry (LC/MS-MS) in the same animals but within extracts of the brain regions showing a strong neuronal transduction of the Slc6a8-FLAG protein: posterior lobules 9 and 10 of cerebellum as well as medulla oblongata (Figures 1 and 2B). As above by ^1^H-MRS, a very strong decrease of Cr was observed in non-injected mKI compared to mWT (in [μmol/g tissue]/cerebellum, 2.04 vs. 10.98 at 5 weeks PI, −81%; 2.04 vs. 10.32 at 14 weeks PI, −80%/medulla, 2.23 vs. 6.70 at 5 weeks PI, −67%; 1.94 vs. 7.04 at 14 weeks PI, −72%) (Figure 2C). Very interestingly, a partial but significant correction of brain Cr was observed in both brain regions of injected mKI compared to non-injected mKI (in [μmol/g tissue]/cerebellum, 2.71 vs. 2.04 at 5 weeks PI, +32%; 2.60 vs. 2.04 at 14 weeks PI, +27%/medulla, 2.74 vs. 2.23 at 5 weeks PI, +23%; 2.36 vs. 1.94 at 14 weeks PI, +22%; Figure 2C), nevertheless remaining far below the mWT levels. The supplementation of injected mKI with Cr, from 5 to 14 weeks PI, did not lead to a further increase in brain Cr (Figure 2C).

### Locomotor activity is rescued in *AAV2/9-2YF-Slc6a8*-injected mKI

**Figure 3:**
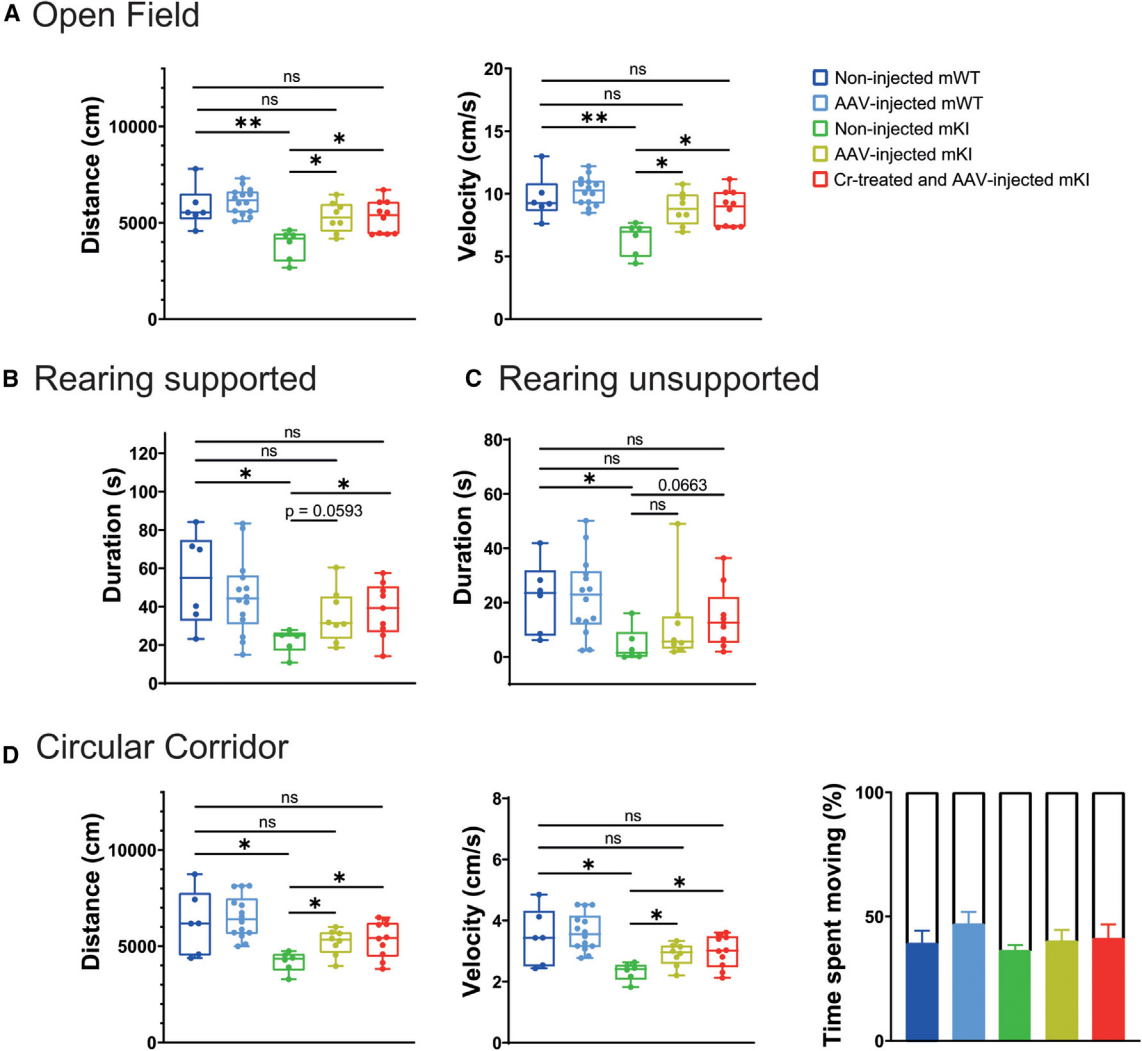
Motor function is rescued in *AAV2/9-2YF-Slc6a8*-injected mKI In OF test (A), distance and velocity were tracked automatically, while rearing supported (B) and unsupported (C) were scored manually. In CC test (D), distance, velocity, and percentage of time spent moving versus not moving were tracked automatically. Six non-injected mWT, 14 *AAV2/9-2YF-Slc6a8*-injected mWT, six non-injected mKI, eight *AAV2/9-2YF-Slc6a8*-injected mKI, and 10 Cr-supplemented and *AAV2/9-2YF-Slc6a8*-injected mKI. Tukey or Mann-Whitney tests after two-way ANOVA; ns, not significant; ∗*p* < 0.05, ∗∗*p* < 0.01.

Sixty percent of CTD patients present motor dysfunction.^15^ We showed recently that our *Slc6a8*^*Y389C*^ rat CTD model presents mild impaired motor function as well as reduction of muscular mass and thinner myocytes.^33^^,^^34^ We thus analyzed whether motor activity was improved in injected mKI. Motor function was evaluated with open-field (OF) and circular corridor (CC) tests, as well as by scoring of rearing (standing up on hind limbs, a spontaneous behavior requiring muscle performance, coordination, and stability for its execution; rearing “supported” if one or both superior limbs are used for support, or “unsupported” when no superior limb used). Non-injected mKI moved significantly less distance with less velocity in both OF and CC (Figures 3A and 3D) and tended to spend less time moving in CC (Figure 3D). Moreover, non-injected mKI presented a significant decrease in rearing supported and unsupported (Figures 3B and 3C), as already shown.^34^ In comparison, injected mKI, with or without Cr supplementation, moved significantly more distance with more velocity in OF and CC as compared to non-injected mKI, recovering the levels of mWT rats (Figures 3A and 3D). In CC, injected mKI, with or without Cr supplementation, also tended to spend more time moving (Figure 3D). Injected mKI showed a near-significant recovery in rearing supported (Figure 3C) and a tendency of increase for rearing unsupported (Figure 3D) compared to non-injected mKI. Finally, Cr-supplemented and injected mKI showed significant rescue in rearing supported (Figure 3C) and near-significant rescue in rearing unsupported (Figure 3D) compared to non-injected mKI. Injected mWT did not show any behavioral difference as compared to non-injected mWT (Figure 3).

### Rescued myocytes in *AAV2/9-2YF-Slc6a8*-injected mKI

**Figure 4:**
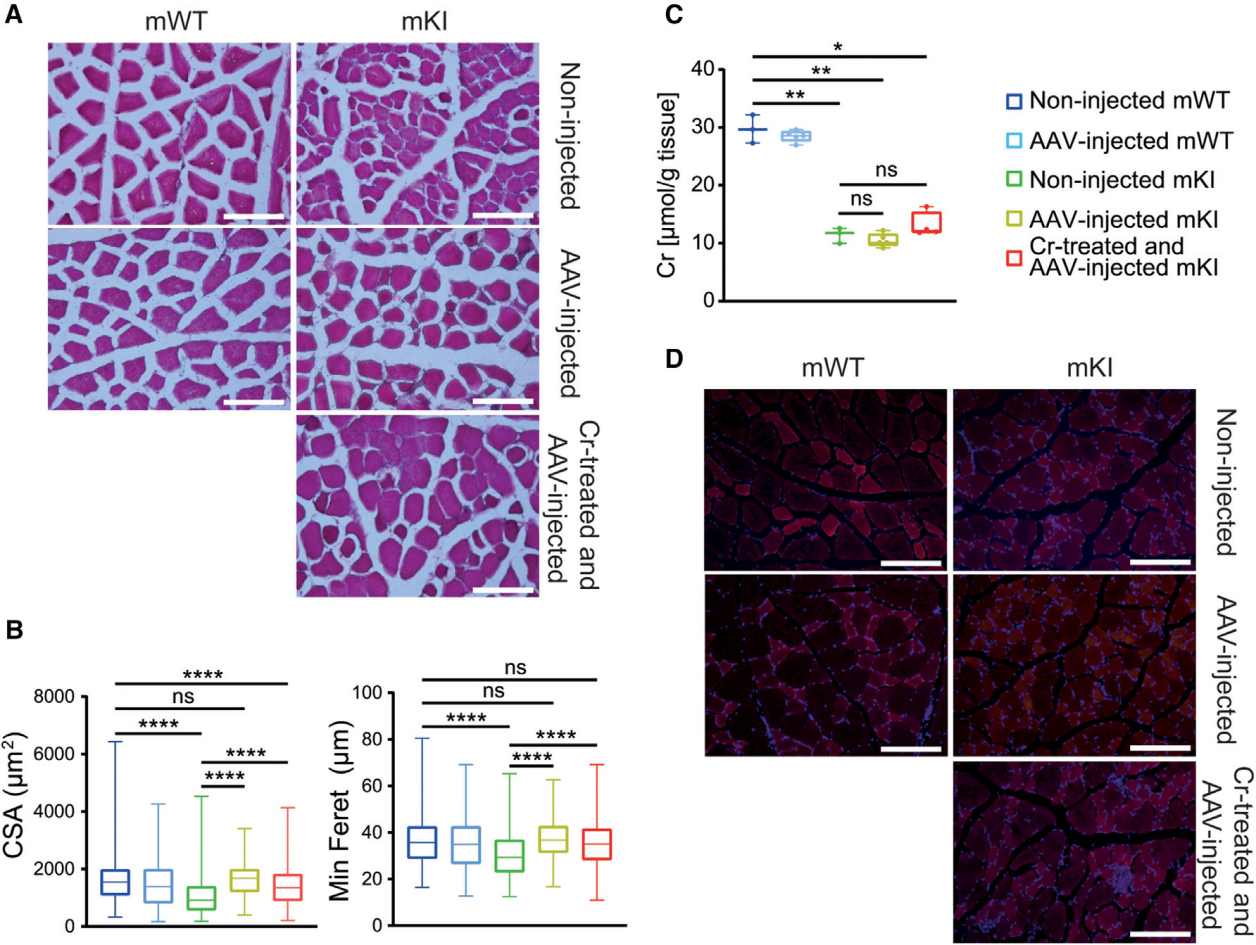
Rescued myocytes in *AAV2/9-2YF-Slc6a8*-injected mKI (A) Representative hematoxylin-eosin staining in transversal section of quadriceps muscle of IC *AAV2/9-2YF-Slc6a8*-injected mWT and mKI. (B) Quantifications of myocytes minimum Feret diameter and cross-sectional area per each group. (C) No Cr recovery in muscle of IC-injected mKI. (D) Absence of transduction of Slc6a8-FLAG Cr transporter in myocytes of IC-injected rats. Anti-FLAG immunofluorescence (red). The only red signal is due to autofluorescence of myocytes (with type I slow-twitch fibers autofluorescing more than type II fast-twitch fibers; see non-injected mWT panel in particular). Three non-injected mWT, three *AAV2/9-2YF-Slc6a8*-injected mWT, three non-injected mKI, three *AAV2/9-2YF-Slc6a8*-injected mKI, and three Cr-supplemented and *AAV2/9-2YF-Slc6a8*-injected mKI. For minimum Feret and cross-sectional area: 340–442 measurements per mWT groups and 410–564 measurements per mKI groups. Tukey test after two-way ANOVA; ns, not significant; ∗*p* < 0.05, ∗∗*p* < 0.01, ∗∗∗∗*p* < 0.0001.

Since motor function depends not only on CNS but also on muscle, and as muscle is home of about 80% of total body Cr, we analyzed the quadriceps muscles of the *Slc6a8*^*Y389C*^ rats under our gene-therapy strategy. As previously shown,^34^ myocyte minimum Feret diameter and cross-sectional area (CSA) from non-injected mKI were significantly reduced in comparison with those of non-injected mWT at 14 weeks PI (Figures 4A and 4B). In contrast, injected mKI, with or without Cr supplementation, presented rescued and thicker myocytes as compared to non-injected mKI (Figure 4A), with minimum Feret diameter and CSA at the same level as non-injected mWT rats (Figure 4B). Intramuscular Cr showed a strong reduction in non-injected mKI compared to mWT, while Cr was not rescued in the quadriceps muscles of IC-injected mKI in comparison to non-injected mKI (Figure 4C; with only a non-significant tendency of increase in injected mKI supplemented with Cr), most probably due to absence of Slc6a8-FLAG transduction in myocytes of injected mKI (Figure 4D). No change in the levels of creatine kinase (CK) or CK-MB was observed either (data not shown). As for behavioral tests, injected mWT did not show any difference in their myocytes as compared to non-injected mWT (Figures 4A–4D).

### Urinary and blood markers of CTD, and absence of Slc6a8-FLAG peripheral transduction

**Figure 5:**
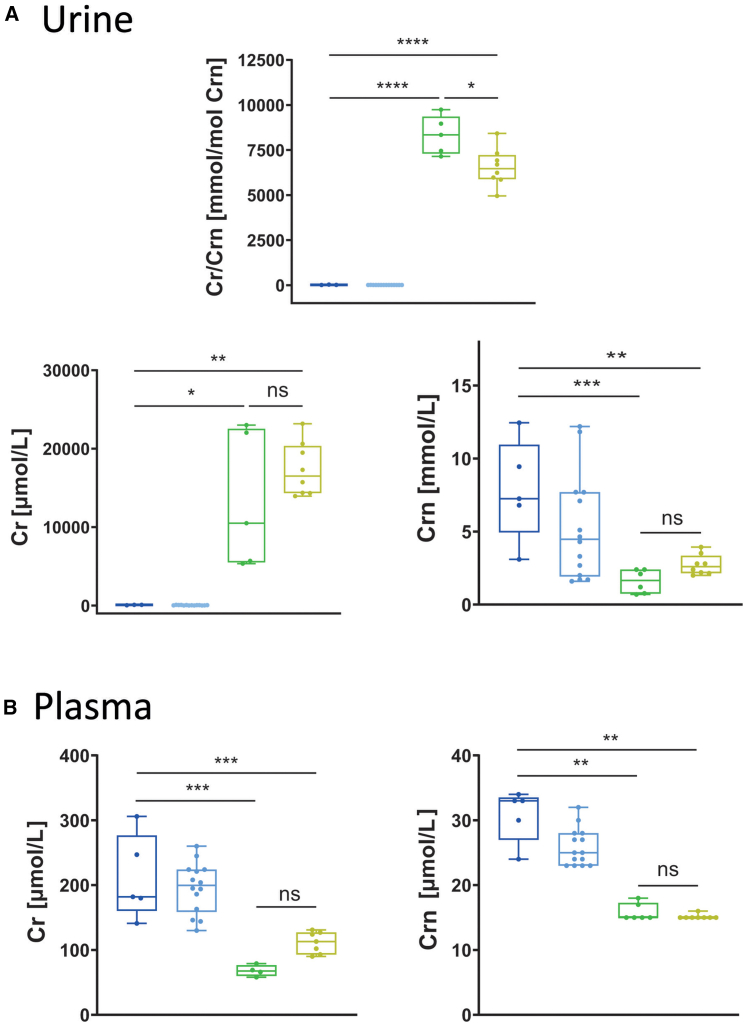
Urinary and blood Cr and Crn in *AAV2/9-2YF-Slc6a8*-injected mKI rats (A) Urinary Cr/Crn ratio (mmol/mol Crn), Cr (μmol/L) and Crn (mmol/L), and (B) plasma Cr and Crn (μmol/L) in non-injected mWT and mKI, as well as *AAV2/9-2YF-Slc6a8*-injected mWT and mKI, 14 weeks PI. Five non-injected mWT, 14 *AAV2/9-2YF-Slc6a8*-injected mWT, six non-injected mKI, and eight *AAV2/9-2YF-Slc6a8*-injected mKI. Tukey test after two-way ANOVA; ∗*p* < 0.05, ∗∗*p* < 0.01.

One of the markers of CTD is the increase of urinary Cr/Crn ratio, which is also observed in the *Slc6a8*^*xY389C/y*^ mKI^33^ (and this study, Figure 5A: 8340 mmol/mol in mKI vs. 7 mmol/mol in mWT). We show that, at 14 weeks PI, injected mKI presented a significant decrease of Cr/Crn urinary ratio (6466 vs. 8340 mmol/mol; Figure 5A) compared to non-injected mKI. As also shown previously,^34^ non-injected mKI presented a significant decrease of urinary Crn compared to non-injected mWT (1.6 vs. 7.3 mmol/L; Figure 5A). The Cr/Crn ratio decrease was not due to the reactivation of a functional Cr transporter in renal tubules, as no transduction of Slc6a8-FLAG could be observed in kidneys of injected mKI (data not shown), but rather to the tendency of increase of both Cr and Crn (Figure 5A), with Crn increasing more than Cr in injected mKI compared to non-injected mKI (ratio of 1.63 and 1.26 respectively).

While plasma Cr is decreased in non-injected mKI compared to mWT^33^^,^^34^ (and this study, Figure 5B: 68 μmol/L in mKI vs. 182 μmol/L in mWT), we show that injected mKI did not present any significant difference in plasma Cr and Crn as compared to non-injected mKI (Figure 5B). This suggests that IC injection of the *AAV2/9-2YF* vector did not lead to the transduction of peripheral tissues, as observed in muscle (Figure 4D), kidney (see above), and liver (data not shown).

Injected mWT did not show any significant difference with non-injected mWT for urinary Crn, Cr, and Cr/Crn ratio or for plasma Cr and Crn (Figures 5A and 5B).

## Discussion

Among CDS, CTD is the most frequent, with no satisfactory treatment so far.^21^^,^^22^ Here, we describe a new strategy of AAV-driven gene therapy for CTD, through IC injection of a *AAV2/9-2YF-Slc6a8-FLAG* vector in our *Slc6a8*^*Y389C*^ CTD rat model. Injected mKI showed widespread neuronal transduction of the Slc6a8-FLAG protein in cerebellum, medulla oblongata, and spinal cord, together with a partial recovery of Cr in these brain regions. Very interestingly, the functional Slc6a8-FLAG transporter transduced in hindbrain structures and spinal cord allowed the rescue of locomotor activity and myocytes in injected mKI.

### AAV2/9-2YF-driven transduction of Slc6a8-FLAG in posterior CNS

We observed a strong neuronal transduction of Slc6a8-FLAG in posterior cerebellum, pons/medulla oblongata, and spinal cord. While *AAV2/9-2YF* has been demonstrated to transduce both neurons and glial cells in previous studies,^38^^,^^39^ astrocytes, oligodendrocytes, microglia, or MCECs did not appear to be transduced in our experiments.

In posterior lobules 9 and 10 of cerebellum, the most transduced neurons were Purkinje cells. This is of particular interest, as Purkinje neurons are known to highly express CrT and require constantly high levels of Cr,^40^^,^^41^^,^^42^ making them potentially more vulnerable to Cr deficiency. We have shown that Purkinje neurons of our *Slc6a8*^*Y389C*^ mKI rats have decreased density and size of dendritic spines, suggesting that they might present less excitatory inputs and lower synaptic strength, resulting in decreased signaling. As Purkinje cells are the only output neurons projecting from cerebellar cortex and contribute to motor function and coordination, this may participate to CTD motor dysfunction.^34^ The restoration of a functional Cr transporter in their dendritic tree may thus contribute to the induced rescue of myocytes and locomotion demonstrated in this study.

In medulla oblongata as well as in most layers of spinal cord, injected mKI also showed a strong transduction in neurons including their dendrites and axons. Moreover, axons of dorsal (peripheral sensory neurons) and ventral (motoneurons) horns of spinal cord were also transduced. Again, these results suggest that the transduction of Slc6a8-FLAG in the tracts between hindbrain and muscles may also contribute to the restored myocytes and locomotion.

### Partial recovery of Cr in the hindbrain

Most probably due to the proximity of IC injection point, we observed a strong neuronal transduction of the Slc6a8-FLAG protein in hindbrain (posterior cerebellum, medulla oblongata) and spinal cord, while only rare, isolated Slc6a8-FLAG-positive cells were observable in forebrain and anterior/superior lobes of cerebellum. Consequently, no replenishment of Cr was observed within the “classical” brain regions measured by ^1^H-MRS (hippocampus and dorsal lobules 6 and 7 of cerebellum). In contrast, a partial recovery of Cr was achieved in the *AAV*-transduced posterior lobules 9 and 10 of cerebellum as well as in medulla oblongata. One of the functions of the cerebellar posterior lobes is to influence the initiation, planning, and coordination of movement, and to determine the strength, direction, and scope of movement.^43^ It is important to note that, although significant, the recovery of Cr in posterior cerebellum and medulla was very partial and did not reach the mWT levels. This is probably due to the “dilution effect” of the Cr measure in a whole extract of brain regions in which only a low proportion of neurons was transduced. It is reasonable to think (but can only be speculated) that Cr recovery within individual AAV-transduced neurons could reach mWT levels. This very partial recovery of cerebellar and medullar Cr may thus be sufficient to rescue locomotion and myocytes.

The absence of Cr recovery in forebrain and dorsal lobules of cerebellum also suggests that, despite partial restoration of Cr in hindbrain, Cr does not seem efficiently distributed to the other parts of CNS through parenchymal extracellular space. When confirmed, this observation may be very important for the treatment of CTD patients, gene therapy thus necessitating efficient transduction within the whole brain.

Finally, while partial Cr recovery was observed in transduced regions of cerebellum and medulla oblongata, no further increase of Cr could be achieved under combination with Cr supplementation. This is most probably due to the absence of Slc6a8-FLAG transduction in MCECs of BBB, which was expected, *AAV9* efficiently crossing BBB but having a poor tropism of transduction for MCECs.^44^ More work is also needed here to also target MCECs, which, in contrast to the astrocytic feet surrounding BBB, do express Slc6a8 physiologically.^40^^,^^45^

Altogether, our findings suggest that IC *AAV2/9-2YF*-induced transduction of the Slc6a8-FLAG Cr transporter led to a very partial but sufficient recovery of Cr in cerebellum, medulla oblongata, and spinal cord, which allowed the restoration of an otherwise CTD-induced impaired neuronal activity in these brain-to-muscle pathways.

### Rescue of locomotion and myocytes

Cr is essential for muscle strength and performance, and motor function depends on coordinated muscle and brain functions. Reduced muscular mass and myocyte diameter associated with low Cr content leads to decreased muscle performance, in turn affecting motor function.^46^ As published in CrT KO mice^24^^,^^25^^,^^26^ and our *Slc6a8*^*Y389C*^ rat,^34^ motor functions can be impaired in *in vivo* CTD models, as observed also in 60% of CTD patients.^15^^,^^16^ Their most prevalent symptom is hypotonia, also with signs of spasticity, coordination dysfunction, and dystonia.

Our gene-therapy approach transducing the functional Slc6a8-FLAG protein showed, with or without Cr supplementation, the rescue of locomotion and myocytes, but without concomitant increase of intramuscular Cr.

Active stimulation of myocytes by motor neurons is essential for the correct development of muscle fibers.^47^^,^^48^ We have recently shown in the *Slc6a8*^*Y389C*^ rat that myocytes of mKI present a thinner cross-section compared to mWT and that this is probably due to a developmental rather to a degenerative process.^34^ Indeed, while many neuromuscular disorders are due to impairment of the neuromuscular junctions,^49^ CTD appears different and does not present with affected neuromuscular junctions.^34^

The rescue of myocytes and locomotion occurred without concomitant transduction of Slc6a8-FLAG in myocytes or recovery of intramuscular Cr. Therefore, our data suggest that the *AAV*-transduced functional Slc6a8-FLAG transporter and partial recovery of Cr in posterior CNS, all along the tracts from brain to motoneurons and within motoneurons themselves, are able to protect and support the normal-appearing development of muscles within IC-injected mKI.

In a more general view, our results also suggest that only a very partial recovery of Cr within CNS may be sufficient to correct CTD.

### Conclusions

This new *AAV2/9-2YF-Slc6a8*-driven gene-therapy strategy for CTD, performed through IC injection in the *Slc6a8*^*Y389C*^ rat, led to the resolution of some of the pathophysiological phenotypes observed in this same CTD model as well as in CTD patients: partial rescue of brain Cr and rescue of locomotor activity and myocytes. As IC injection of the vector only allowed Slc6a8-FLAG transduction in the rear part of CNS (cerebellum, medulla oblongata, and spinal cord), more work is needed to improve those CTD phenotypes associated with forebrain structures.

AAV gene therapy provides a great hope for numerous rare genetic diseases, including those affecting CNS,^50^^,^^51^^,^^52^^,^^53^ for which no other satisfactory option of treatment has been identified so far. This first *AAV*-driven strategy thus represents an encouraging advancement for the several developed approaches to treat CTD.^21^

## Materials and methods

### Cloning and production of an *AAV2/9-2YF* vector transducing the rat Slc6a8 transporter

The AAV plasmid was pTR-UF2 (a kind gift from Dr Sergei Zolotukhin, University of Florida)^54^ in which the NeoR expression cassette was deleted, while the mCherry sequence, under control of the CMV promoter and an SV40 polyadenylation sequence, was replaced by the functional open reading frame of the rat *Slc6a8* Cr transporter (GenBank: NM_017348) followed by an eight-amino-acid FLAG sequence (DYKDDDDK) allowing the specific immunodetection of the AAV-transduced Slc6a8_FLAG Cr transporter (clone ID: ORa12376, GenScript, Piscataway, NJ), followed by a woodchuck hepatitis virus (WHP) post-transcriptional regulatory element (WPRE). *AAV* plasmids with the same genetic construction but with the open reading frames of the reporter fluorescent proteins EGFP or mCherry were used as controls. In order to produce the recombinant *AAV2/9-2YF* viral vectors (chosen as known to better cross BBB and more efficiently transduce the brain tissue),^55^ the vector plasmid was encapsidated into a mutant serotype 9 capsid, pXR9-2YF. Briefly, HEK-293T cells (30 10-cm plates) were co-transfected using polyethylenimine (PEI; Sigma-Aldrich, Germany) at a 5:1 (v/w) PEI:DNA ratio and a 2:3:5 molar ratio of vector plasmid, pAdhelper plasmid (Stratagene, USA) and *pAAVXR9-2YF* packaging plasmid expressing the *rep* gene from AAV serotype 2 and the *cap* gene from AAV serotype 9 harboring two mutations of surface tyrosines (tyrosine to phenylalanine mutations, a kind gift from Dr Deniz Dalkara, Institut de la Vision, INSERM, Paris)^56^ with addition of a *Slc6a8*-specific small interfering RNA (siRNA) (30 nM, 5′-CAG GAA AGA UCG UGU ACU UCT-3′) in order to increase the vector yield as previously described.^57^ Fifty hours post transfection, the medium was discarded, and cells were harvested by low-speed centrifugation and resuspended in Tris 50 mM, EDTA 1 mM pH 8.5, NaCl 0.1 M. After five cycles of freezing/thawing, the cellular lysate was clarified by 20-min centrifugation at 11,000 rpm, treated with benzonase (50 units/mL; Sigma-Aldrich, Germany) at 37°C for 30 min, and centrifuged at 11,000 rpm for 20 min to eliminate the residual debris. The virus was further purified by iodixanol step gradient and microconcentrated through Amicon Ultra 15-mL 100K (Merck Millipore, Germany).

Viral genomes (vg) were titrated by quantitative PCR using primers located in inverted terminal repeats (ITR) sequences (forward primer, 5′-GGAACCCCTAGTGATGGAGTT-3′; reverse primer, 5′-CGGCTTCAGTGAGCGA-3′)^58^ and yielded 8 × 10^12^ vg/mL.

The plasma-membrane localization of the expressed Slc6a8-FLAG transporter and its functionality to take up Cr were verified through transfection of HEK293 cells followed by anti-FLAG immunofluorescence as well as through transfection of mKI primary fibroblasts incubated with Cr (Figure S4, including supplemental methods within Figure S4).

### Rat housing and experimentation

Rats were maintained under a 12 h/12 h light-dark cycle. Food devoid of Cr (Safe-150; Safe Diets, France) and water were available *ad libitum*. All experiments were performed with the approval of the veterinary authorities of the Canton de Vaud (Switzerland; authorization VD-3284) in accordance with the regulations of the Swiss Academy of Medical Science and followed the ARRIVE Guidelines 2.0. Efforts were made to minimize stress and number of animals used. Thirty-four Sprague-Dawley mWT and 39 *Slc6a8*^*Y389C/y*^ mKI rats were used in this study as previously described.^33^^,^^34^

Eleven-day-old mKI and mWT rats were injected by direct intracisternal (IC) injection of 10 μL of *AAV2/9-2YF-Slc6a8-FLAG* viral suspensions (10^13^ vg/kg), with the following described protocol.^59^ No shaving of the injection area was needed, as P11 rat pups still do not bear a dense fur. The non-anesthetized pups were held in the hand, and the injection site was swabbed and sterilized with 70% EtOH, also allowing a better visibility. No incision was made in the scalp, and direct IC injection was performed through the injection point determined as the “upside-down V” shape at the basis of the rat skull located just above cisterna magna, using a 32-gauge BD Micro-fine Insulin syringe (Becton Dickinson NJ USA, no. 32486). A guard made of a sticky tape at 2 mm from the tip of the needle was placed to avoid a too-deep injection that could kill the animal. No leakage of cerebrospinal fluid (CSF) was observed at the injection site, and *AAV2/9-2YF* virus injection did not induce any mortality, all injected rats living up to the end of the experiments and showing no sign of distress. Animals were sacrificed at 5 and 14 weeks PI to assess *AAV2/9-2YF* transduction efficacy and cellular tropism. Starting from 5 weeks PI, a subgroup of injected mKI were exposed to daily Cr supplementation (Cr monohydrate, Sigma-Aldrich, St. Louis, MO) in the drinking water (2 g/kg/day). *In vivo* brain ^1^H-MRS scans were performed at 5 and 14 weeks PI. Behavioral tests were performed at 12 weeks PI; to avoid potential detrimental effect of circadian rhythms on behavioral performances, all behavioral tests were performed in the morning (9–11 a.m.).

### *In vivo*^1^H-MRS

*In vivo* 9.4T ^1^H-MRS was performed on a Horizontal Actively Shielded 9.4 T system (Magnex Scientific, Oxford, UK) interfaced to a Varian Direct Drive console (Palo Alto, CA, USA) as previously described in hippocampus and cerebellum of mWT and non-injected mKI, as well as injected mKI plus or minus Cr supplementation.^33^

### Tissue and liquid collection

Animals were sacrificed at two different time points for histological and biochemical analyses: 5 and 14 weeks PI to investigate respectively short-term and long-term efficacy of transduction of the Slc6a8 transporter. Animals were anesthetized with 4% isoflurane under 70% compressed air and 30% O_2_ in order to collect plasma and urine. Rats were placed in supine position and abdominal cavity was opened to collect urine from the bladder. Finally, the thoracic cavity was opened, blood was collected transcardially in microtubes with lithium heparin (Sarstedt, Germany) and centrifuged at 2,000 × *g* for 5 min. Supernatant (plasma) was transferred in a new tube. Plasma and urine were stored at −80°C for further analysis. Then, brain, muscle (quadriceps), and spinal cord were rapidly dissected out, rinsed in ice-cold phosphate-buffered saline (PBS), and prepared for biochemical or histological analysis. Tissues for biochemical analysis were immediately frozen (liquid nitrogen) then stored at −80°C. Tissues for histological analysis were fixed in 4% paraformaldehyde (PFA) in PBS overnight at 4°C, then rinsed with PBS and sunk in 18% (muscle) or 30% (brain and spinal cord) sucrose before being embedded in Tissue-Tek (O.C.T. Compound, Sakura Finetek, USA), frozen, and stored at −80°C.

### Blood and urine, as well as brain and muscular extracts, analysis

Measurements of Cr in blood and urine as well as in brain and muscle tissue extracts were performed by LC-MS/MS as described previously.^33^^,^^34^ Measurement of Crn in blood and urine, as well as CK and CK-MB, was performed on a COBAS 8000 automate (Roche, Switzerland).

### Histology

Transverse 16-μm- (brain and muscle) or 30-μm-thick (spinal cord) cryosections were cut with a cryostat (Leica CM3050, Leica BioSystems, Switzerland), mounted on microscope slides, and frozen at −80°C until use for immunofluorescence.

### Immunofluorescence

To stain the sections, slides were tempered for 5 min at room temperature (RT), incubated with 4% PFA-PBS (15 min RT), washed (PBS, 3 × 5 min), and proceeded for immunofluorescence. Before the blocking step, an antigen retrieval was performed with sodium citrate buffer (pH = 6.0) for 20 min at 100°C and 20 min at RT. Slides were washed one time with PBS and two times with PBS and 0.01% Triton X-100 (Fluka, France) for 5 min. For non-specific binding site blocking, slides were incubated 1 h at RT with 1% bovine serum albumin (BSA, Sigma-Aldrich, Germany) in PBS and 0.01% Triton X-100. Primary (1:100 or 1:250, overnight 4°C) and secondary (1:500, 1 h RT) antibodies are listed below. At the end of immunolabeling, slides were incubated with diamidino-2-phenylindole (DAPI; Invitrogen, USA) (5 min 1:5000 in PBS), washed (PBS 3 × 5 min), and mounted with Anti-Fade Fluorescence Mounting Medium (Abcam, UK). All primary antibodies used in this study are commercially available, as follows: FLAG (1:500, A00187, GenScript, USA), GFAP (1:100, MAB360, Merck, Germany), NeuN (1:100, MAB377, Merck, Germany), MAP2 (1:250, 4542, Cell Signaling Technology, USA), pNFM (1:500, MAB5254, Merck, Germany), and MBP (1:250, MAB386, Merck, Germany). Secondary antibodies were goat anti-mouse or anti-rabbit and donkey anti-goat immunoglobulin (Ig) G labeled with Alexa Fluor 488 (green) or 568 (red) (Life Technologies, USA).

### Hematoxylin-eosin staining

Slides were tempered (15 min RT), immersed in distilled water, dehydrated in increasing concentrations of ethanol (until 100%), and rehydrated to distilled water before staining. Slides were incubated for 5 min in eosin, washed with water, incubated for 2 min in hematoxylin, washed with water, dehydrated in increasing concentrations of ethanol (until 100%), incubated for 3 min in xylol, mounted in Eukitt (BioSystems, Switzerland), and dried for 24 h.

### Imaging and quantifications

Sections were photographed using a Hamamatsu Nanozoomer S60 microscope (Hamamatsu Photonics, Japan) with 20× objective allowing for multiple magnification through pixel binning, and by a Zeiss 780 laser scanning microscope (Carl Zeiss, Germany) using a Zeiss Plan-Apochromat, 20×/0.8 NA objective and 40×/1.3 NA oil objective. Images were analyzed with NDP.view2 software and ZEN software. Images of stained muscles were taken on an Olympus BX50 microscope (Olympus Life Science, Japan). Muscle cross-sectional areas and minimum Feret diameter were obtained with ImageJ software.

### Behavioral tests

#### OF

OF test was conducted to evaluate locomotor activity and exploratory behaviors. Rats were introduced in a round open arena (1-m diameter) with black walls and floor. The light was adjusted to a level of 10 lx in the center of the arena. Animals were placed close to the wall of the arena, and OF activity was tested for a 10-min period. Average velocity, total distance moved, and time spent moving or not moving (considering thresholds of 2 and 1.75 cm/s, respectively) were calculated from the same recordings using EthoVision XT tracking software (Noldus, the Netherlands). Grooming, rearing supported, and unsupported behaviors were hand scored blindly with the Observer XT software (Noldus, the Netherlands), and cumulative duration and frequency of such behaviors were analyzed.

#### CC

Rats were individually placed for 30 min in a black acrylic CC with external and internal diameters of 50 and 40 cm, respectively, and a height of 40 cm. Average velocity, total distance moved, and time spent moving or not moving (considering thresholds of 2 and 1.75 cm/s, respectively) were calculated using EthoVision XT tracking software.

### Statistical analysis and graphs

Statistical analysis was performed with GraphPad Prism software (Prism 9 for Windows; GraphPad Software, San Diego, CA). If not stated otherwise, results are presented as mean values ± standard deviations. In box-plots, the horizontal line within the box represents the median; the whiskers represent the range of the data (minimum to maximum). Two-way ANOVA was performed with *post hoc* Tukey or non-parametric Mann-Whitney tests (comparison of three or more groups for two independent variables); a two-tailed *p* < 0.05 was considered statistically significant, with *p* < 0.05 (∗), *p* < 0.01 (∗∗) and *p* < 0.0001 (∗∗∗∗).

## Data and code availability

The raw data required to reproduce the above findings are available upon request.

## Supplemental information

Document S1. Figures S1–S4

Document S2. Article plus supplemental information
