# Supplementary material for: Rescue of myocytes and locomotion through AAV2/9-2YF intracisternal gene therapy in a rat model of creatine transporter deficiency
Source: Mol Ther Methods Clin Dev. 2024 Apr 23;32(2):101251. doi: 10.1016/j.omtm.2024.101251 (PMC11091509; doi:10.1016/j.omtm.2024.101251)
Supplement: Document S1. Figures S1–S4 [file mmc1.pdf]

OMTM, Volume *32*

Supplemental information

Rescue of myocytes and locomotion through *AAV2/9-2YF* intracisternal gene therapy in a rat model of creatine transporter deficiency

Gabriella Fernandes-Pires, Marcelo Duarte Azevedo, Marc Lanzillo, Clothilde Roux-Petronelli, Pierre-Alain Binz, Cristina Cudalbu, Carmen Sandi, Liliane Tenenbaum, and Olivier Braissant

Supplemental material

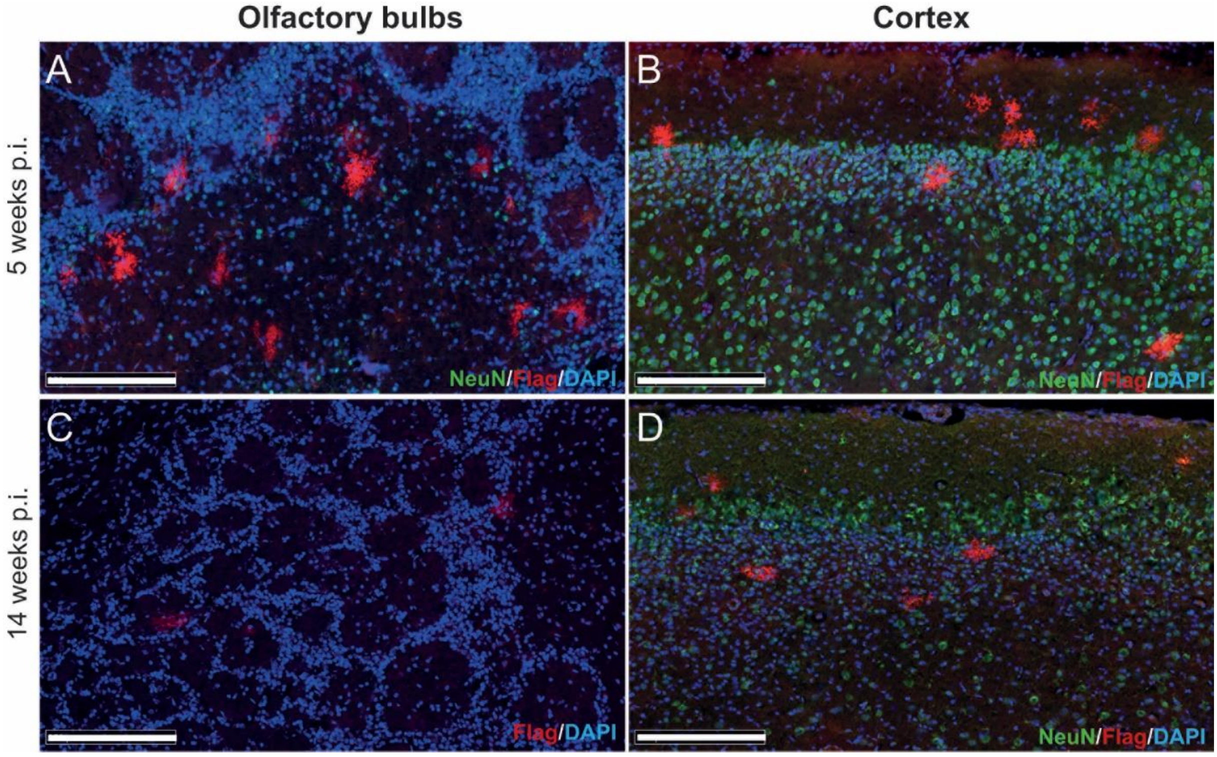

<u>Figure S1</u>: Transduction of the Slc6a8-Flag protein in forebrain (olfactory bulbs and cortex) of *AAV9-Slc6a8*-injected mKI rats, at 5 and 14 weeks post-injection (PI).

Representative images of immunostaining for Slc6a8-Flag in olfactory bulbs (**A**/**C**) and cortex (**B**/**D**) at 5 (**A**/**B**) and 14 (**C**/**D**) weeks PI. Slc6a8-Flag in red; NeuN in green; DAPI in blue. Bar scale = 250 µm.

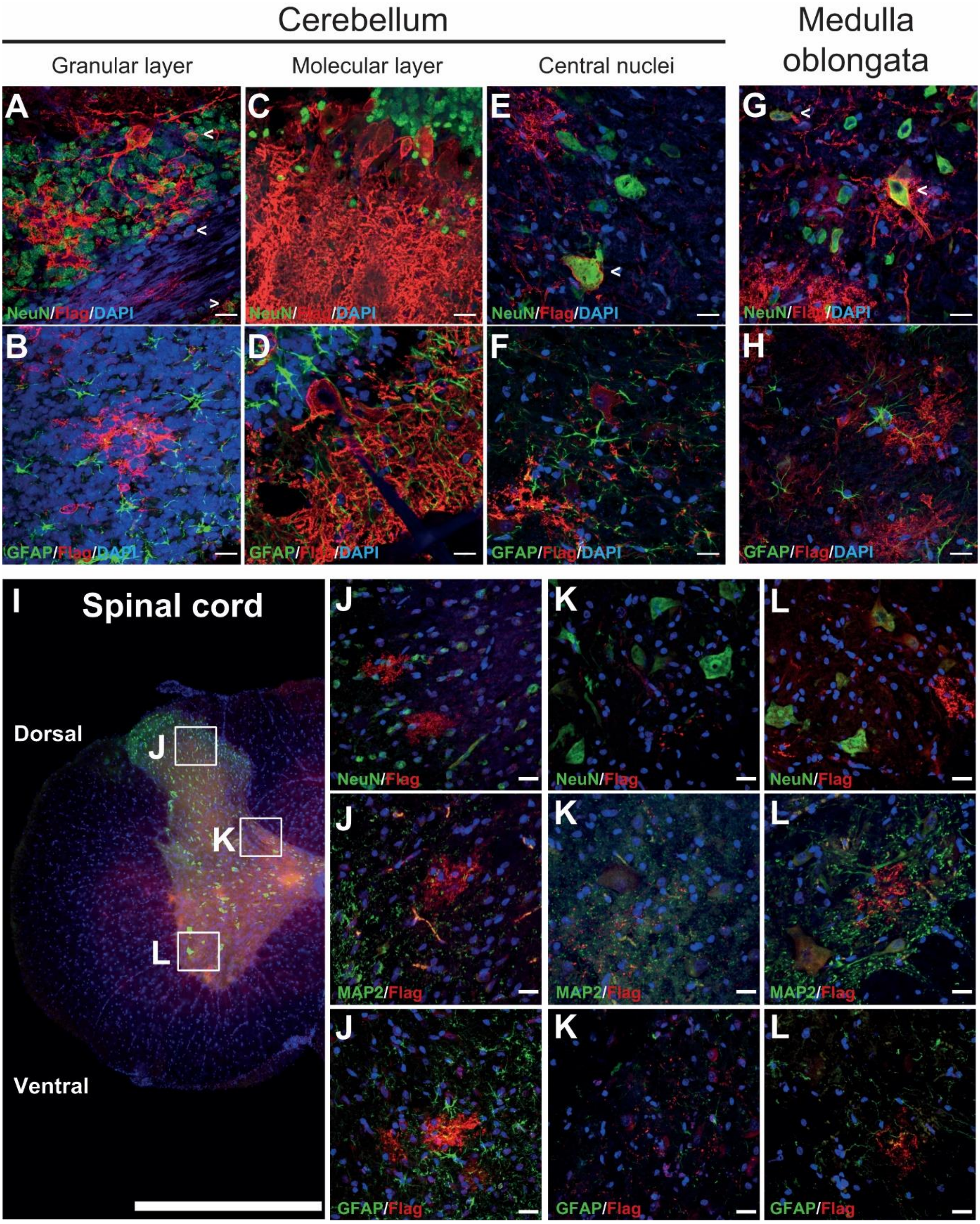

<u>Figure S2</u>: Transduction of the Slc6a8-Flag protein in CNS of *AAV2/9-2YF-Slc6a8-Flag*-injected mKI rats, at 5 weeks PI.

Representative images of immunostaining for Slc6a8-Flag in different region of the brain: (**A**/**B**) granular layer of cerebellum, (**C**/**D**) molecular layer of cerebellum, (**E**/**F**) cerebellar nuclei and (**G**/**H**) medulla oblongata. (**I**) Cross-section of the spinal cord, with (**J**) lamina I/II, (**K**) lamina IV/V, and (**L**) lamina IX. Slc6a8-Flag in red; NeuN, GFAP or MAP2 in green; DAPI in blue. Bar scale for overview of spinal cord = 250 µm. Bar scale for details of cerebellum, medulla and spinal cord = 20 µm.

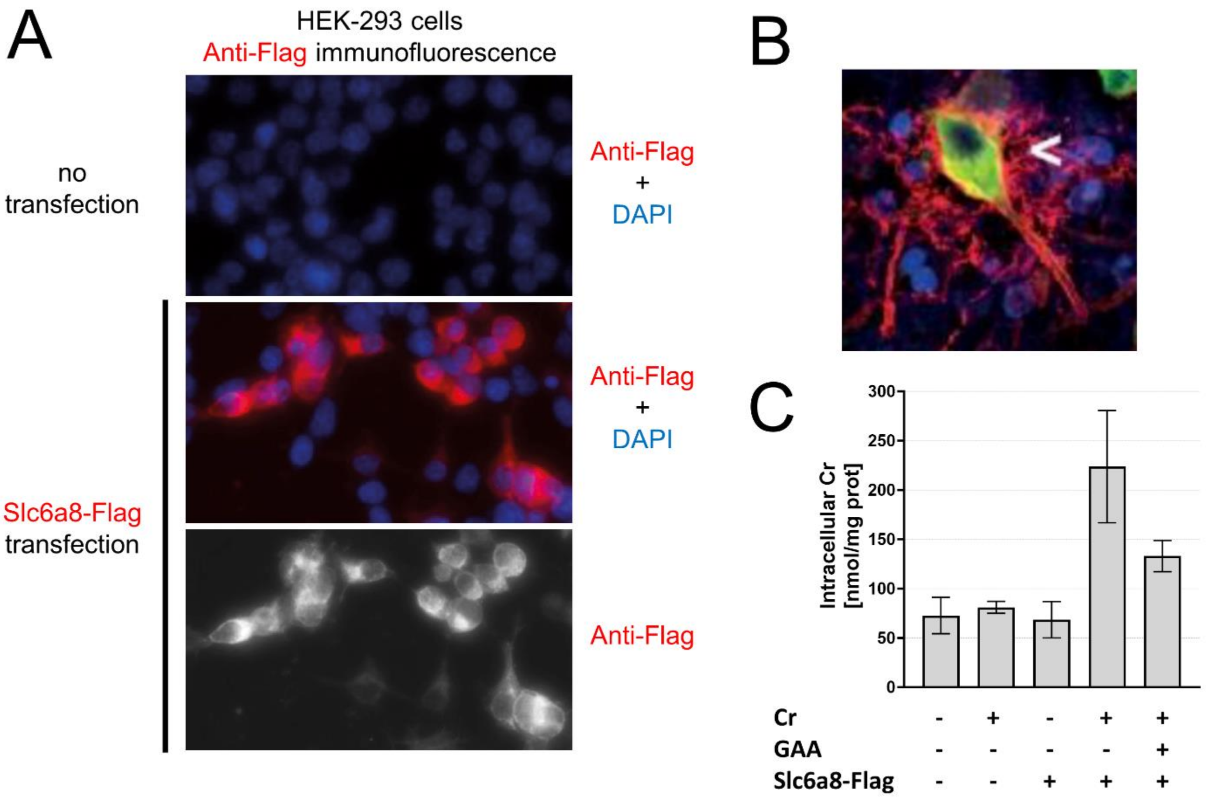

<u>Figure S3</u>: Plasma membrane localization of the transduced Slc6a8-Flag protein, and its functionality as creatine transporter.

(**A**) HEK293 cells transfected with the *pTR-CMV-Slc6a8-Flag* expression plasmid, showing the expression of the Slc6a8-Flag transporter (red/white) on the cell surface. Immunofluorescence episcopy; DAPI in blue. (**B**) Confocal enlargement of **Figure S2G**, showing the plasma membrane localization of Slc6a8-Flag on a cerebellar neuron. Slc6a8-Flag: red; NeuN: green; DAPI: blue. (**C**) Cr transporter-deficient *Slc6a8^{Y389C}* mKI primary fibroblasts transfected with the *pTR-CMV-Slc6a8-Flag* expression plasmid, demonstrating that *pTR-CMV-Slc6a8-Flag* transfection leads to the expression of a functional creatine transporter on the fibroblasts plasma membrane. In absence of Cr in culture medium and of *pTR-CMV-Slc6a8-Flag* transfection, a basal level of intracellular Cr is observed, due to the expression of AGAT and GAMT by fibroblasts (1st lane). This basal level does not change after incubation with Cr alone (Cr transporter-deficient fibroblasts; 2nd lane) or with *pTR-CMV-Slc6a8-Flag* transfection alone (3rd lane). *pTR-CMV-Slc6a8-Flag* transfection combined with Cr incubation leads to an important increase of intracellular Cr (4th lane), which can be competed by guanidinoacetate (GAA) co-incubation (5th lane).

Supplemental methods for Figure S3:

For A: HEK293 cells were cultured in 6-well plates up to 80% confluence, then transfected with *pTR-CMV-Slc6a8-Flag* (10 µg/well / lipofectamine protocol Gibco 31985062). 48h after transfection, cells were fixed (4% paraformaldehyde) and observed by anti-Flag immunofluorescence.

For C: Cr transporter-deficient fibroblasts were isolated from the tail of a *Slc6a8^{Y389C}* mKI and cultured in 6-well plates up to 80% confluence. Some were transfected with *pTR-CMV-Slc6a8-Flag* (10 µg/well / lipofectamine). 24h after transfection, Cr uptake was evaluated after 4h incubation with 200 µM Cr plus or minus 1 mM GAA for competition, followed by two washes of ice-cold PBS, cell extraction and measure of intracellular Cr by LC/MS-MS as described (Braissant et al 2010, Neurobiol Dis 37:423-433). N=3 per condition; mean values ± standard deviation.

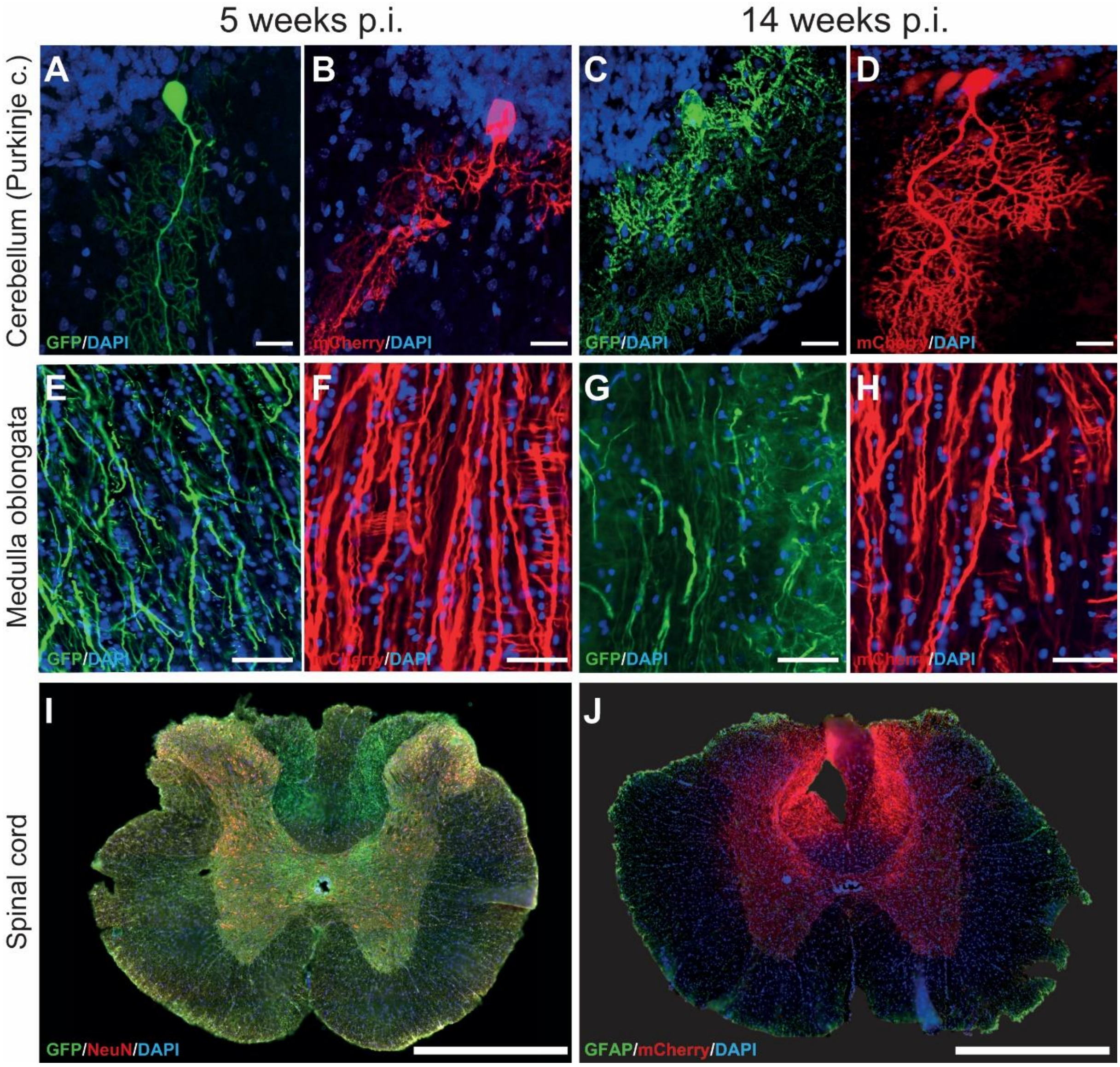

<u>Figure S4</u>: Transduction of fluorescent reporter proteins EGFP and mCherry in CNS of *AAV2/9-2YF-EGFP*- and *AAV2/9-2YF-mCherry*-injected mWT rats.

Representative images of immunostaining for EGFP and mCherry 5 and 14 weeks PI, in different region of the brain: (**A-D**) cerebellum (Purkinje cells), (**E-H**) medulla oblongata, (**I/J**) spinal cord. GFP (**A/C/E/G/I**) or GFAP (**J**) in green, mCherry (**B/D/F/H/J**) or NeuN (**I**) in red; DAPI in blue. Bar scale for details of cerebellum and medulla oblongata = 100 µm. Bar scale for overview of spinal cord = 1 mm.
